# Systematic review with meta-analysis: Efficacy and safety of biological treatment on salivary gland function in primary Sjögren’s syndrome

**DOI:** 10.3389/fphar.2023.1093924

**Published:** 2023-02-14

**Authors:** Xiaoyan Wang, Xiang Lin, Yingying Su, Hao Wang

**Affiliations:** ^1^ Department of Stomatology, Beijing Tiantan Hospital, Capital Medical University, Beijing, China; ^2^ Department of Biochemistry and Molecular Biology, School of Basic Medicine, Capital Medical University, Beijing, China; ^3^ School of Chinese Medicine, The University of Hong Kong, Hong Kong, China; ^4^ The University of Hong Kong-Shenzhen Institute of Research and Innovation (HKU-SIRI), Shenzhen, China

**Keywords:** meta-analysis, biologics, primary Sjögren’s syndrome, salivary gland function, efficacy, safety

## Abstract

**Objective:** The study aimed to assess the efficacy and safety of clinical trials of biologics in improving the salivary gland (SG) function in primary Sjögren’s syndrome (pSS), which has not been analyzed critically and systematically.

**Methods:** PubMed, Web of Science, ClinicalTrials.gov, the EU Clinical Trials Register, and the Cochrane Library were searched for clinical trials that reported effects of biological treatment on the SG function and safety in pSS patients. Inclusion criteria were defined following participants, interventions, comparisons, outcome, and study design (PICOS) recommendations. The objective index (the change of unstimulated whole saliva (UWS) flow) and the serious adverse event (SAE) were assessed as main outcome measures. A meta-analysis of the efficacy and safety of the treatment was conducted. Quality assessment, sensitivity analysis, and publication bias were assessed. The effect size together with a 95% confidence interval was used to estimate the efficacy and safety of biological treatment and was plotted as a forest plot.

**Results:** The literature search yielded 6,678 studies, nine of which fulfilled the inclusion criteria, with seven randomized controlled trials (RCTs) and two non-RCT clinical studies. Generally, biologics do not significantly increase UWS from the baseline of pSS patients compared to the control group at a matched time point (*p* = 0.55; standard mean difference, SMD = 0.05; 95% confidence interval, CI: −0.11 and 0.21). However, pSS patients with shorter disease duration (≤3 years; SMD = 0.46; 95% CI: 0.06 and 0.85) were prone to have a better response to biological treatment by showing higher increased UWS than patients with longer disease duration (> 3 years; SMD = −0.03; 95% CI: −0.21 and 0.15) (*p* = 0.03). For the meta-analysis of the safety of biological treatment, the SAEs in the biologics group were significantly higher than those of the control group (*p* = 0.0021; log odds ratio, OR = 1.03; 95% CI: 0.37 and 1.69).

**Conclusion:** Biological intervention during the early course of the disease may benefit pSS patients better than that during the late course. Significantly, more SAEs in the biologics group indicate that the safety of biologics needs to be addressed for future biological clinical trials and treatment.

## Introduction

Primary Sjögren’s syndrome (pSS) is a systematic and chronic autoimmune disease with a strong organ bias, which is clinically manifested with exocrine gland dysfunction (particularly dry mouth and dry eyes) and various extra-glandular manifestations. Serological and histopathological assessments show an increased level of serum autoantibodies and lymphocytic infiltration in exocrine glandular tissues, respectively (Bowman, 2018). pSS is among the most common autoimmune rheumatic diseases, with the prevalence ranging from 0.01% to 3% of the general population (Maciel et al., 2017).

Compelling evidence suggests many biological processes are involved in the pathogenesis of pSS and have contributed to the establishment of salivary gland (SG) pathology. In the earlier phase of disease progression, the innate immune response and CD4^+^ T cells play a more important role (Nocturne and Mariette, 2018; Verstappen et al., 2021). In the later stages of pSS, both the innate immune system and the activated T cells can induce the activation of B cells (Nocturne and Mariette, 2018; Verstappen et al., 2021) and, thereafter, establish the positive feedback loop in pSS. During these biological processes, a group of cytokines (e.g., TNFα, IFNs, IL-1, IL-2, and IL-6) produced by involved immune cells and SG epithelial cells also play pivotal roles in this immunopathological process (Retamozo et al., 2018).

Hyposalivation significantly affects the life quality of pSS patients, who suffer from sicca (dry mouth) symptoms with various complaints, including dental caries, change of taste, and difficulties in eating, sleeping, and speaking (Wang et al., 2020a). Saliva is produced by SG acinar cells. Watery and mucous-rich saliva, from serous and mucous acinar cells, respectively, is transferred to ductal cells and finally to the oral cavity (Pringle et al., 2019). The saliva is mainly produced by major SGs, i.e., the parotid gland, the sublingual gland, and the submandibular gland, together with a small amount of saliva being produced by minor SGs (Proctor, 2000). The SG is an epithelial organ; its homeostasis is maintained by SG progenitor cells, by generating several epithelial cell types, ductal, acinar, and myoepithelial cells (Wang et al., 2021).

The mechanism behind deteriorated SG function remains elusive. Thus, current treatment for pSS mainly focuses on symptom alleviation, such as using artificial saliva and secretagogues (e.g., pilocarpine and cevimeline). Debates exist as to whether chronic inflammation results in hyposalivation in pSS patients and whether the depletion of inflammation contributes to the alleviation of a dry mouth (Jonsson et al., 1993; Soto-Rojas and Kraus, 2002; Mignogna et al., 2005; Nocturne and Mariette, 2013). Recent studies have found a significant association between infiltration and the senescence of the SG epithelial progenitor cell niche, indicating the prolonged effect of lymphocytic infiltration on SG epithelial cells and their function (Wang et al., 2020b).

Biologics are medical products produced through the biological process, working as a new type of drug that suppresses immune responses and reduces inflammation (Singh et al., 2011; Chong et al., 2020). Biologics are increasingly being used in treating autoimmune disorders, such as systemic lupus erythematosus and rheumatoid arthritis, and pSS (Goodman, 2015; Nocturne et al., 2016). In pSS, a series of biological clinical trials aiming to intervene in the immunobiological process of pSS have been administered and completed, with targets including cytokines, and the activation and proliferation of B cells or T cells (Table 1). Although SG function has been shortlisted as one of the primary outcomes in drug screening tests and clinical trials, currently, the efficacy and safety of biologics on SG hypofunction improvement have been found to vary. Additionally, several clinical trials examining biological treatment in pSS have been completed in the most recent three years (St Clair et al., 2018; Baer et al., 2020; Felten et al., 2020), which have not been (systematically) reviewed. Thus, systematically analyzing these biological clinical trials may provide new insights into whether SG hypofunction is attributed to inflammatory factors or certain types of them. Herein, we conducted a systematic meta-analysis to investigate their efficacy and safety in pSS patients.

**TABLE 1:** Molecular target of included biologics trials in pSS.

Biologic	Molecular target	Target effect
Rituximab	CD20	Inhibits the proliferation of stimulated B cells
Abatacept	CD80 and CD86	Inhibits T-cell activation
Baminercept	LTαβ	Blocks lymphoid tissue organization and chronic inflammation
Infliximab	TNFα	Inhibits TNFα
Mizoribine	IMPDH	Inhibits the proliferation of activated B cells
Tocilizumab	IL-6R	Inhibits the binding of IL-6 and IL-6R
Iguratimod	BAFF–BCMA/TACI pathway	Reduces the number of plasma cells and inhibits the production of IgG
Seletalisib	PI3Kδ	Reduces the accumulation of B and T lymphocytes and plasma cells

## Methods

### Data sources and search strategy

Electronic databases of PubMed, Web of Science, ClinicalTrials.gov, the EU Clinical Trials Register, and the Cochrane Library were searched for this systematic review and meta-analysis (updated until October 2022). Studies about the biological treatment of pSS with endpoints of the alleviation of hyposalivation and the safety of drugs were searched. Studies being searched were limited to those published in English. The keywords used were primary Sjögren’s syndrome, biologics, saliva, and safety or serious adverse event (SAE) and additional terms such as rituximab, abatacept, seletalisib, iscalimab (CFZ533), infliximab, etanercept, BMS-931699/Lulizumab, and epratuzumab. The title, abstract, and full text were downloaded and read for being assessed for relevance. The reference list of included articles was examined to include the possible missing studies. Published or registered clinical trials with results were considered as the source of this systematic analysis.

### Study selection (inclusion criteria and exclusion criteria)

The selection of studies was conducted according to the participants, interventions, comparisons, outcome, and study design (PICOS) recommendations.

### Inclusion criteria

Study type: Original research. Participants (P): pSS patients. Interventions (I): Biological treatment. Comparisons (I): No intervention and interventions without biological treatment, standard treatment, or placebo. Outcome (O): The change of saliva secretion and the occurrence of SAE during/after the biological treatment. Study design (S): Well-designed clinical trials.

### Exclusion criteria

Study type: Review articles, case reports, comments, and articles with the abstract only. Incomplete data or data impossible to be extracted were excluded. Replicative studies or studies using the same group of patients were excluded. Participants (P): Non-pSS patients (e.g., secondary SS patients) and non-human animals. Interventions (I): Non-biological treatment. Comparisons (I): Absence of comparisons. Outcome (O): No outcome related to the change of saliva secretion or SAE during/after the biological treatment. Study design (S): Non-clinical trials.

### Data extraction

All searched articles were screened by two independent authors for determining whether the inclusion and exclusion criteria were met. The discrepancy was resolved by discussion with the third author. Data on the author; year of publication; country/region where the trial was conducted; sample size; type and dosage of biological intervention; age, gender, and disease duration of participated patients; the method of measurement of saliva secretion, outcomes for the biological treatment, and control groups were extracted. The change of unstimulated whole saliva (UWS) secretion from baseline was used for the evaluation of salivary gland function. Total SAEs and SAEs specified into different system disorders were employed for the safety assessment of biologics.

### Assessment of study quality

The quality of RCT studies was assessed by using the Cochrane risk of bias tool by two independent authors and illustrated by Review Manager 5.3 (Liang et al., 2022). When inconsistencies occur, the third author will participate in the discussion for the assessment. The Cochrane tool is composed of five domains, namely, the randomization process, deviations from the intended interventions, missing outcome data, measurement of the outcome, and selection of the reported result, with each domain being judged as the low risk of bias, high risk of bias, or some concerns. The quality of the non-RCT trial was assessed by the Methodological Index for Non-Randomized Studies (MINORS) scoring system (Slim et al., 2003). The MINORS scoring system contains eight domains (a clearly stated aim, inclusion of consecutive patients, prospective collection of data, endpoints appropriate to the aim of the study, unbiased assessment of the study endpoint, a follow-up period appropriate to the aim of the study, loss to follow-up less than 5%, and prospective calculation of the study size) together with additional criteria (an adequate control group, contemporary groups, and baseline equivalence of groups) in the case of a comparative study, with the score of 0, 1, or 2.

### Assessment of sensitivity analysis and publication bias

The sensitivity analysis of the UWS response and SAEs in the biological treatment and control groups was conducted by omitting the included studies one by one. The publication bias is shown by the funnel plot and also quantified by the regression-based Begg’s test for small-study effects.

### Statistical analysis

The extracted data were imported into Stata 16.0 software and subjected to statistical analysis. For dichotomous variables, a log odds ratio (OR) with 95% confidence interval (CI) was calculated. For continuous outcomes, standard mean differences (SMDs), as indicated, with 95% CI were calculated. To assess the heterogeneity among studies, *p*- and *I* ^*2*^ values were calculated. When *p* ≥ 0.10 and *I* ^*2*^ ≤ 50%, suggesting the homogeneity was appropriate for meta-analysis, the fixed effects model was employed. Otherwise, a random effects model was used. In addition to the overall assessments, these included studies were also subjected to two groups by the mean disease duration (less or more than 3 years) for subgroup analysis to assess the effect of biologics and their safety.

## Results

### Identification of studies

**FIGURE 1:**
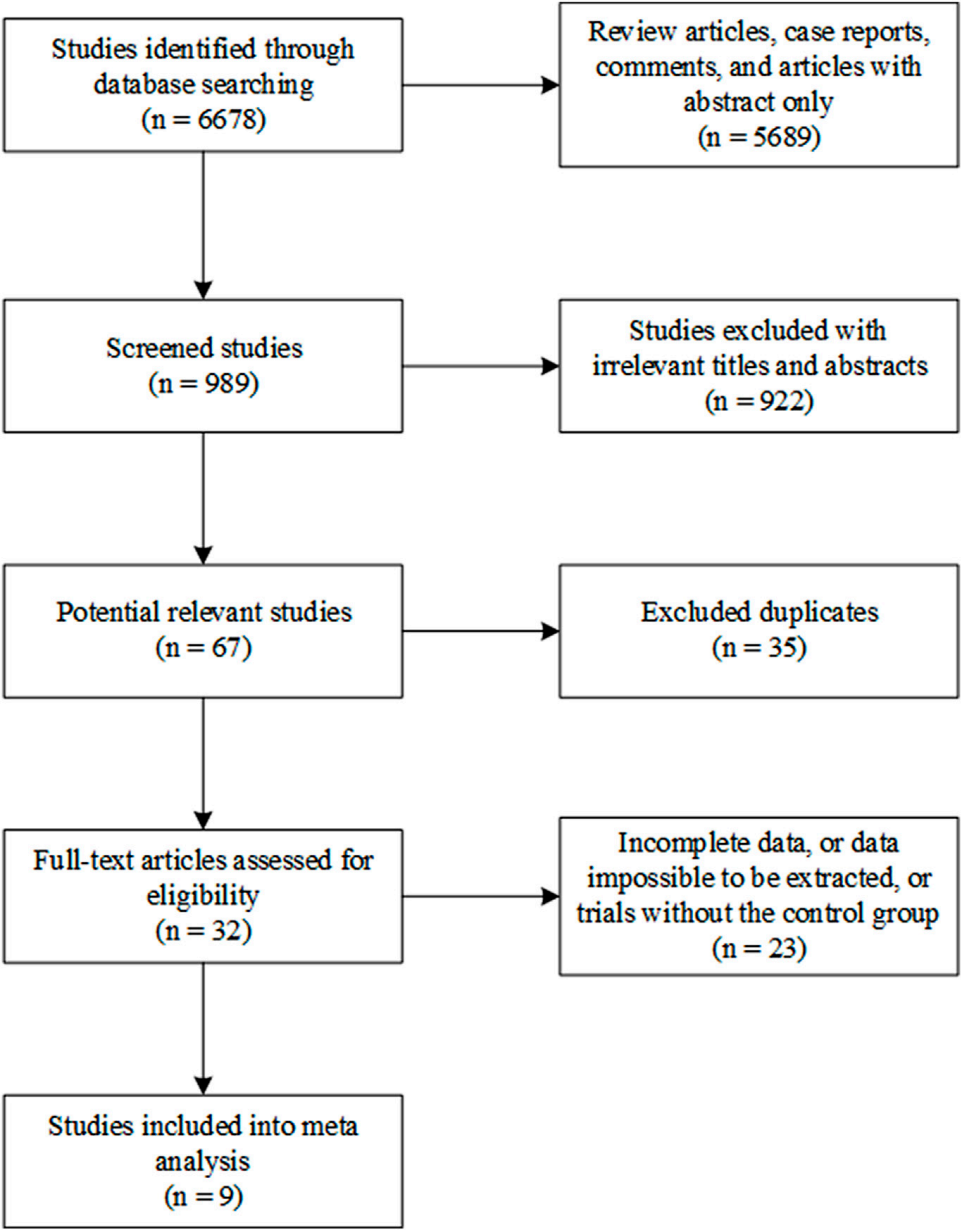
Flow chart of study selection including inclusion and exclusion criteria.

From database searching, 6,678 studies were identified. Of them, 5,689 review articles, case reports, comments, and articles with only the abstract were excluded. A total of 922 irrelevant studies were excluded from this systematic analysis after title and abstract reading. A total of 35 studies were excluded because of duplication. The full text of the remaining 32 studies was read, and 23 of them were found to contain incomplete data or data that were impossible to be extracted or were studies that did not contain the control group. Finally, nine studies were included in this systematic meta-analysis (Figure 1) (Mariette et al., 2004; Nakayamada et al., 2009; Meijer et al., 2010; Carubbi et al., 2013; St Clair et al., 2018; Baer et al., 2020; Felten et al., 2020; Juarez et al., 2021; Shao et al., 2021).

### Characteristics of included studies

In these nine clinical trials, with seven RCTs (the clinical trial registration number is indicated in Supplementary Table S1) and two non-RCT trials, the sample size ranged from 27 to 187 patients; the effect of eight biologics on the change of the UWS flow and SAE was investigated and compared with the control group (Table 2). Of the control group among these nine studies, seven employed a placebo, one used DMARDs, and one took the untreated group, as described in Table 2. The molecular target of included biologics is shown in Table 1. Studies of Meijer, Carubbi, Baer, Mariette, Nakayamada, and Shao had one or more measurement time points, including week 24 (Mariette et al., 2004; Meijer et al., 2010; Carubbi et al., 2013; Baer et al., 2020; Shao et al., 2021). Except for the study performed by Mariette et al. (2004), which measured the change of UWS of pSS patients at weeks 10 and 22, and the study performed by Juarez et al. (2021), which measured at time points of weeks 8 and 12, the outcome of week 24 [week 22 was selected for the study of Mariette et al. (2004), and week 12 was selected for the study of Juarez et al.(2021)] was selected as the time point for this systematic meta-analysis.

**TABLE 2:** Characteristics and the change of unstimulated whole saliva (UWS) flow changes and the serious adverse event (SAE) of included studies.

Author	Year	Biologics vs. control	Experimental group				Control group		Experimental group		Control group	
			Disease duration (mean, years)	UWS change (mean ± SD, ml/min)	Total (n)	Disease duration (mean, years)	UWS change (mean ± SD, ml/min)	Total (n)	SAE	Total (n)	SAE	Total (n)
Meijer	2010	Rituximab vs. placebo	5.3	0.05 ± 0.31	20	5.6	0.02 ± 0.12	10	0	20	0	10
Carubbi	2013	Rituximab vs. DMARDs	1.2	0.22 ± 0.24	19	1.1	0.02 ± 0.24	22	0	19	0	22
Baer	2020	Abatacept vs. placebo	5.0	0.05 ± 0.69	81	5.1	0.11 ± 0.69	87	9	92	3	95
St. Clair	2018	Baminercept vs. placebo	N.R.	0.06 ± 0.17	33	N.R.	0.07 ± 0.17	19	5	33	1	19
Mariette	2004	Infliximab vs. placebo	4.9	0.03 ± 0.15	54	4.0	0.02 ± 0.19	49	6	54	1	49
Nakayamada	2009	Mizoribine vs. untreated	1.9	0.60 ± 2.88	31	2.9	0.00 ± 2.33	28	0	31	0	28
Felten	2021	Tocilizumab vs. placebo	4.4	−0.1 ± 1.63	47	4.9	0.00 ± 1.44	43	14	55	6	55
Shao	2020	Iguratimod vs. placebo	12.8	0.004 ± 0.07	36	10.3	0.002 ± 0.06	19	0	36	0	19
Juarez	2021	Seletalisib vs. placebo	6.1	−0.04 ± 0.11	13	7.6	−0.03 ± 0.09	14	3	13	1	14

N.R., not reported.

### Quality of studies, sensitivity analysis, and publication bias

**FIGURE 2:**
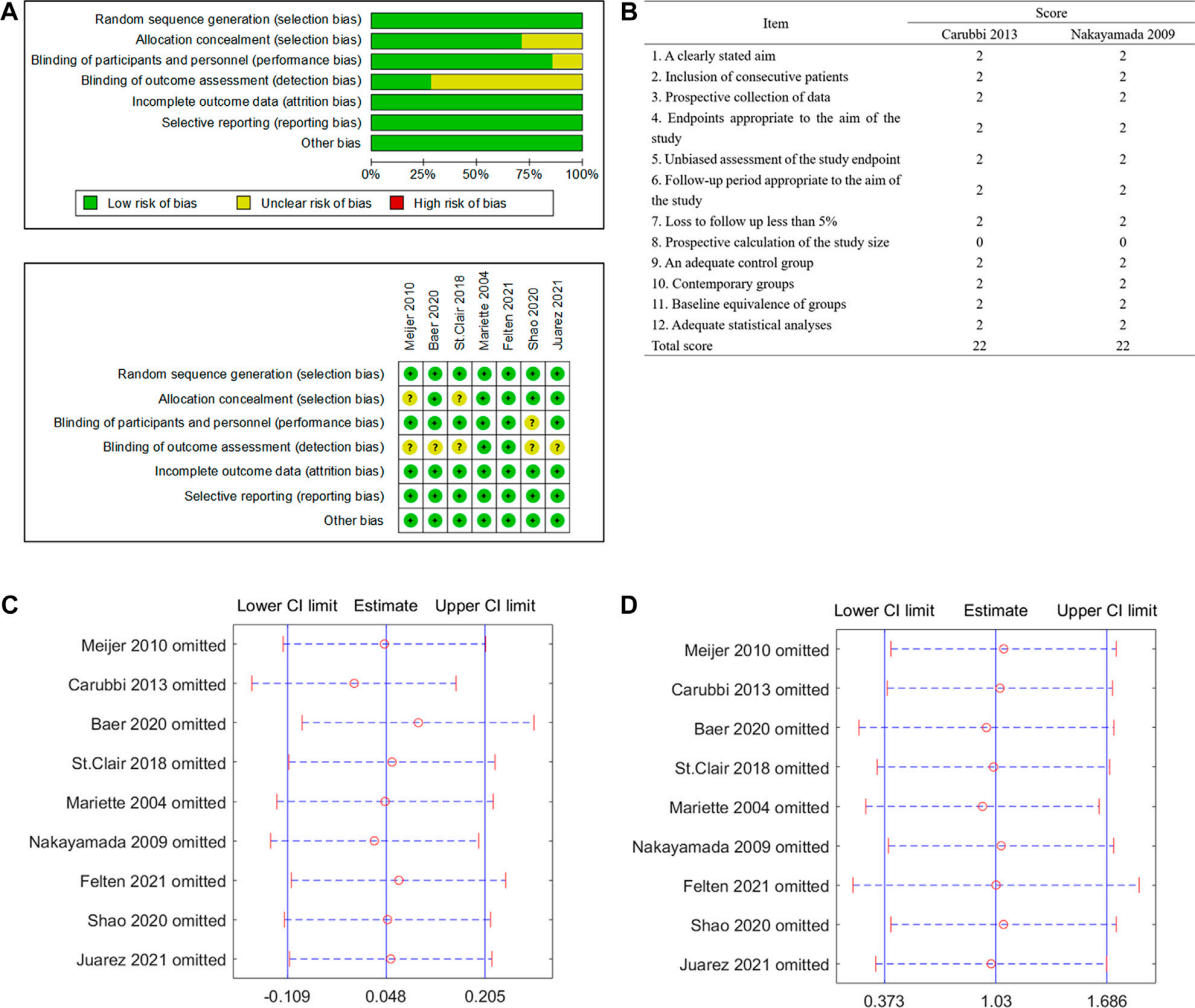
Quality assessment and sensitivity analysis of included studies. **(A)** Randomized controlled trials (RCTs) assessed by the Cochrane risk of bias tool. **(B)** Non-RCT trials assessed by the Methodological Index for Non-Randomized Studies (MINORS) scoring system. **(C)** Sensitivity analysis of the change of unstimulated whole saliva (UWS). **(D)** Sensitivity analysis of the occurrence of serious adverse events (SAEs).

The quality of the seven RCTs assessed by the Cochrane risk of bias tool is shown in Figure 2A. None of the included RCTs were assessed as “high risk of bias.” The quality assessment of the two non-RCT clinical trials was assessed by the MINORS scoring system; both were scored 22 (the total score is 24, Figure 2B).

**FIGURE 3:**
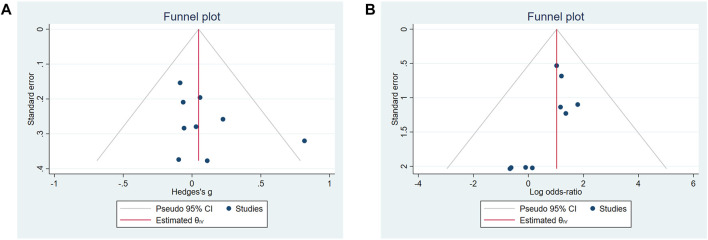
Funnel plots of the publication bias. **(A)** Change of unstimulated whole saliva (UWS). **(B)** Occurrence of serious adverse events (SAEs).

The sensitivity analysis of the UWS response and SAEs in the biological treatment and control groups shows that the overall effect of size did not change significantly, indicating that the included studies did not have extreme conditions, and the heterogeneity was appropriate (Figures 2C, D). No significant publication bias for the change of the UWS flow (Figure 3A, *p* = 0.348) and the outcome of the SAE (Figure 3B, *p* = 1.971) of these included studies was found for this meta-analysis. There was also no significant bias for SAEs specified into different system disorders (Supplementary Table S2).

### Effects of biological treatments on pSS UWS

**FIGURE 4:**
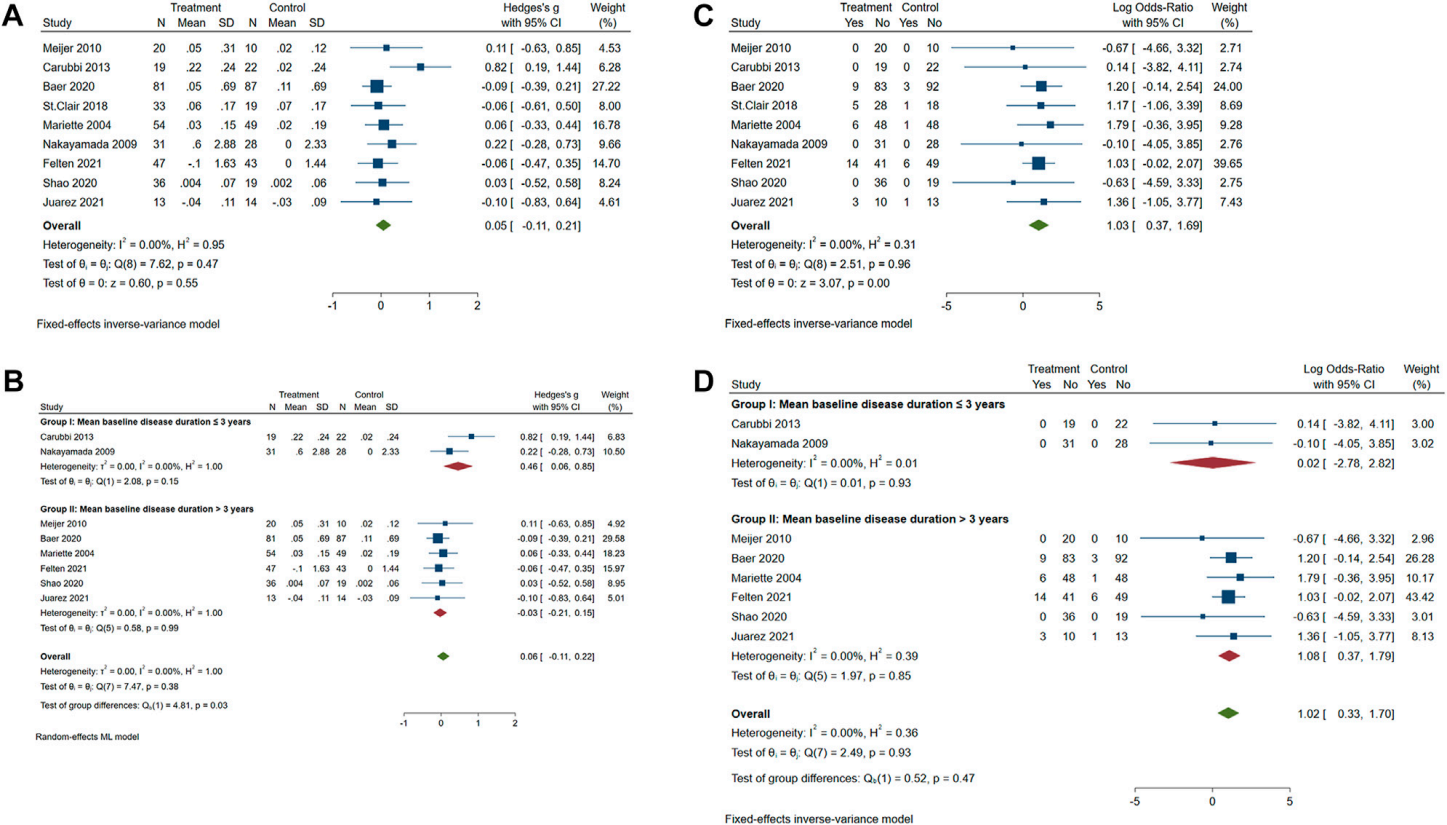
Forest plot of studies comparing the intervention of biologics and control groups on pSS patients. **(A)** Change of the course of disease (UWS). **(B)** Change of UWS when trials were subgrouped by patient baseline disease duration. **(C)** Occurrence of serious adverse events (SAEs). **(D)** Occurrence of SAEs when trials were subgrouped by patient baseline disease duration.

The meta-analysis of the overall effect of biological treatments on the UWS flow in pSS patients is shown in Figure 4A. These nine studies reported acceptable heterogeneity (*p* = 0.47, *I* ^*2*^ = 0.00%), showing no significant effect on UWS of pSS patients (*p* = 0.55; standard mean difference (SMD) = 0.05; 95% CI: −0.11 and 0.21). During these biological clinical trials, early intervention was recommended to expect a better response on pSS treatment (Nakayamada et al., 2009; Carubbi et al., 2013; St Clair et al., 2018). Accordingly, these included studies were subjected into two groups (mean disease duration less or more than 3 years, Figure 4B). As the disease duration in the study of St. Clair et al. is not indicated in the publication (St Clair et al., 2018), the authors contacted St. Clair and made sure the baseline disease duration was not collected during the study. Therefore, this study was not included in the part of the subgrouping meta-analysis. The overall heterogeneity is acceptable (*p* = 0.38 and *I* ^*2*^ = 0.00%). A significant group difference was found (*p* = 0.03). The increase of UWS flow after biological treatment was much greater in patients with shorter baseline disease duration (≤ 3 years; SMD = 0.46; 95% CI: 0.06 and 0.85) than those with longer baseline disease duration (> 3 years; SMD = −0.03; 95% CI: −0.21 and 0.15).

### The treatment of biologics on pSS patients showing significantly more SAEs than the control group

As shown in Figure 4C, SAEs were reported in five studies. A significant difference in SAEs was found between the biologics and control groups (*p* = 0.0021, OR = 1.03, and 95% CI: 0.37 and 1.69), with an acceptable heterogeneity (*p* = 0.96 and *I* ^*2*^ = 0.00%). The disease duration of pSS patients did not cause a difference in the incidence of SAEs between the biologics and control groups (Figure 4D; *p* = 0.47, OR = 1.02, and 95% CI: 0.33 and 1.70). Reported SAEs in the biological treatment group included five neoplasms, six immune system disorders, five infections and infestations, five gastrointestinal disorders, three general disorders, three hepatobiliary disorders, two reproductive system issues, three musculoskeletal and connective tissue disorders, two cardiac disorders, one blood and lymphatic system disorder, and one nervous system disorder (Supplementary Table S3). SAEs reported in the control group included three immune system disorders, two infections and infestations, one gastrointestinal disorder, one general disorder, two musculoskeletal and connective tissue disorders, one cardiac disorder, one blood and lymphatic system disorder, two nervous system disorders, and one respiratory, thoracic, or mediastinal disorder (Supplementary Table S3). To specify the possible disorders that are more closely associated with biological treatment, the meta-analysis of SAEs classified into different systems was performed. There was no significant publication bias for all kinds of SAEs in different system disorders (Supplementary Figure S1). Results showed that the incidence of SAEs of all types of system disorders was not significantly different between the biologics and control groups (Supplementary Figure S2).

## Discussion

In this meta-analysis, currently finished biological clinical trials were systematically analyzed. The biological treatment could not significantly increase SG function at the time point that was chosen to perform the comparison; however, the intervention of biological treatment in the earlier phase of pSS might result in a better response, exhibited with an increased UWS flow.

pSS is a systemic autoimmune disease, characterized by lymphocytic infiltration in epithelial organs and decreasing SG function. However, whether there is a causal relationship between lymphocytic infiltration and undermined SG function (manifested by decreased saliva flow) is still controversial. It was commonly accepted that infiltrated lymphocytes occupy and destroy the parenchyma SG and result in the decrease of saliva production (Du et al., 2021). However, some recent studies revealed there is a poor relationship between saliva flow and the degree of SG infiltration (Jonsson et al., 1993; Soto-Rojas and Kraus, 2002; Mignogna et al., 2005; Wang et al., 2020b). In this meta-analysis, it was found that current clinical trials using various biologics inhibiting the activity of inflammation *via* different targets could not significantly increase the residual saliva production. This indicates the function of damaged SG could not be restored solely by inflammation control. However, this study does not exclude that there might be a therapeutic effect of biological treatment on salivary gland function at other time points which were not possibly analyzed.

Interestingly, in the trial with the biological intervention of pSS patients with shorter disease duration, the increased secretion of the UWS flow of patients was significantly higher than that in patients with longer disease duration. Early diagnosis to recognize pSS, in addition to providing this potential benefit, may also prolong the treatment window available and should be prioritized in future endeavors. However, only two biological trials were performed in early pSS patients; more studies are needed to test the efficacy of biologics in the alleviation of hyposalivation in early pSS.

As introduced earlier, the homeostasis of SG is maintained by the cellular duplication and differentiation of SG progenitor cells (Wang et al., 2021). Our previous study found that SG progenitor cells in pSS patients have hypofunctional proliferation and differentiation abilities, partially resulting from cellular senescence (Pringle et al., 2018; Wang et al., 2020b). It was also found that cytokines and chemokines associated with the chronic immune response in pSS probably promote the process of SG progenitor cell senescence (Pringle et al., 2018). Biological intervention in pSS patients with shorter disease duration causes a better SG response, which may indicate that pSS patients at this phase still contain considerable functional SG progenitor cells. However, with disease progression, in the later phase, when SG progenitor cells have been immersed in the proinflammatory microenvironment for a long time and the cellular senescence has potentially become more established, immunological intervention using biologics may be largely fruitless.

In this meta-analysis, the safety of biological intervention was also considered. Significant, more frequently occurring SAEs remind us that drug safety needs to be carefully considered before intervention. Interestingly, the individual system SAEs were not significantly different between biological treatment and the control groups, indicating the increased SAEs in the biologics group were not confined to one system or one type of disease. Influence factors of the safety of biologics may include the type of biologics, the dose, the administration route, and the administration frequency (van de Kerkhof et al., 2016; Ingrasciotta et al., 2018; Kamata and Tada, 2018). The safety and efficacy need to be highly balanced during biological trials. The treatment plan needs to be adjusted promptly according to the investigation of the treatment efficacy and the occurrence of adverse events.

Blinding of the outcome assessment is a common item that may cause a high risk of bias (five out of seven RCTs). Moreover, the limitation to this study is that only nine trials fulfilled the inclusion criteria and were included for the systematic analysis. If more outcomes are reported with the completion of current ongoing biological trials, the conclusion of this systematic review could be more convincing.

To conclude, although clinical trials of biologics show limited efficacy in the restoration of SG function in pSS patients, the better response of SG function to biologics in pSS patients with shorter disease duration reminds us to administrate timely biological interventions in the early course of the disease before the function of SG becomes irreversible. Additionally, the safety of administered drugs needs to be always kept in mind. Future biological clinical trials need to concentrate on trials that have better SG response and higher safety, such as interventions that can topically, rather than systemically, alleviate the inflammatory microenvironment.

## Abbreviations

pSS, primary Sjögren’s syndrome; SG, salivary gland; UWS, unstimulated whole saliva; SAE, serious adverse event; RCT, randomized controlled trial; PICOS, participants, interventions, comparisons, outcomes, and study design; IFN, interferon; MALT, mucosa-associated lymphoid tissue; RCT, randomized controlled trial; MINORS, Methodological Index for Non-Randomized Studies; OR, odds ratio; CI, confidence interval; SMD, standard mean difference.
