# Supplementary figures and images for: Systematic review with meta-analysis: Efficacy and safety of biological treatment on salivary gland function in primary Sjögren’s syndrome
Source: Front Pharmacol. 2023 Feb 14;14:1093924. doi: 10.3389/fphar.2023.1093924 (PMC9972580; doi:10.3389/fphar.2023.1093924)

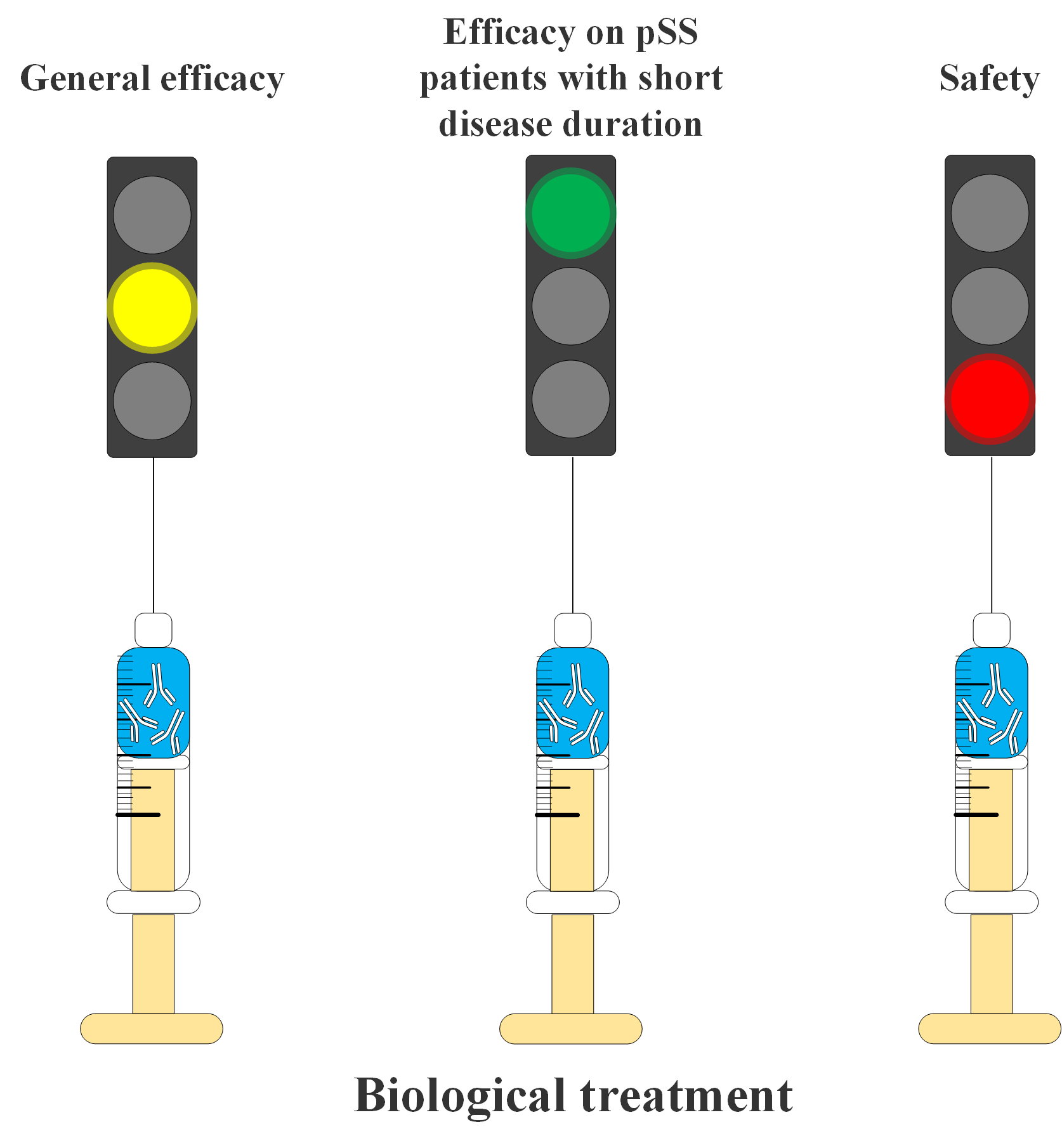

Supplement: Supplementary file 1 [file Image1.TIF]
