# Supplementary material for: Systematic review with meta-analysis: Efficacy and safety of biological treatment on salivary gland function in primary Sjögren’s syndrome
Source: Front Pharmacol. 2023 Feb 14;14:1093924. doi: 10.3389/fphar.2023.1093924 (PMC9972580; doi:10.3389/fphar.2023.1093924)
Supplement: Supplementary file 2 [file DataSheet1.DOCX]

**Systematic review with meta-analysis: efficacy and safety of biological treatment on salivary gland function in primary Sjögren’s syndrome**

Xiaoyan Wang^1,2*^, Xiang Lin^3,4^, Yingying Su^1^, Hao Wang^1^

**Affiliations**

1. Department of Stomatology, Beijing Tiantan Hospital, Capital Medical University, Beijing, China.
2. Department of Biochemistry and Molecular Biology, School of Basic Medicine, Capital Medical University, Beijing, China.
3. School of Chinese Medicine, The University of Hong Kong, Hong Kong, SAR, China.
4. The University of Hong Kong-Shenzhen Institute of Research and Innovation (HKU-SIRI), China.

**Financial support:** This research was supported by National Natural Science Foundation of China (82201084), China Postdoctoral Science Foundation (2022M722232), Miaopu Project of Beijing Tiantan Hospital, Capital Medical University (2023MP10), and General Research Fund, Hong Kong Research Grants Council (27111820 and 17116521).

**Contact Information**

*Corresponding author:

Dr. X. Wang Email: [wangxiaoyan@bjtth.org](mailto:wangxiaoyan@bjtth.org)

**Supplementary table S1. Clinical registration number of included randomized controlled trials (RCT)**

| RCT | Clinical registration number |
| --- | --- |
| Meijer 2010 | NCT00363350 |
| Baer 2020 | NCT02915159 |
| St.Clair 2018 | NCT01552681 |
| Mariette 2004 | N.R. |
| Felten 2021 | NCT01782235 |
| Shao 2020 | ChiCTR1900024642 |
| Juarez 2021 | NCT02610543 |

N.R.: not reported.

**Supplementary table S2. The *p* value of Begg's test for small-study effects**

| SAEs | *p* |
| --- | --- |
| SAE (neoplasms) | 1.998 |
| SAE (immune system disorders) | 1.971 |
| SAE (infections and infestations) | 1.882 |
| SAE (gastrointestinal disorders) | 1.924 |
| SAE (general disorders) | 1.749 |
| SAE (hepatobiliary disorders) | 1.971 |
| SAE (reproductive system issues) | 1.882 |
| SAE (musculoskeletal and connective tissue disorders) | 1.749 |
| SAE (cardiac disorders) | 1.825 |
| SAE (blood and lymphatic system disorders) | 1.398 |
| SAE (nervous system disorders) | 0.917 |
| SAE (respiratory, thoracic and mediastinal disorders) | 1.083 |

**Supplementary table S3. Serious adverse event (SAE) specified into different system disorders of included studies**

| Author | | Meijer | Carubbi | Baer | St.Clair | Mariette | Nakayamada | Felten | Shao | Juarez |
| --- | --- | --- | --- | --- | --- | --- | --- | --- | --- | --- |
| Year | | 2010 | 2013 | 2020 | 2018 | 2004 | 2009 | 2021 | 2020 | 2021 |
| Experimental group (n) | SAE (neoplasms) | 0 | 0 | 1 | 1 | 1 | 0 | 2 | 0 | 0 |
|  | SAE (immune system disorders) | 0 | 0 | 2 | 0 | 1 | 0 | 2 | 0 | 1 |
|  | SAE (infections and infestations) | 0 | 0 | 1 | 0 | 2 | 0 | 2 | 0 | 0 |
|  | SAE (gastrointestinal disorders) | 0 | 0 | 1 | 1 | 0 | 0 | 2 | 0 | 1 |
|  | SAE (general disorders) | 0 | 0 | 0 | 0 | 2 | 0 | 1 | 0 | 0 |
|  | SAE (hepatobiliary disorders) | 0 | 0 | 1 | 2 | 0 | 0 | 0 | 0 | 0 |
|  | SAE (reproductive system issues) | 0 | 0 | 0 | 0 | 0 | 0 | 2 | 0 | 0 |
|  | SAE (musculoskeletal and connective tissue disorders) | 0 | 0 | 2 | 0 | 0 | 0 | 0 | 0 | 1 |
|  | SAE (cardiac disorders) | 0 | 0 | 1 | 0 | 0 | 0 | 1 | 0 | 0 |
|  | SAE (blood and lymphatic system disorders) | 0 | 0 | 0 | 0 | 0 | 0 | 1 | 0 | 0 |
|  | SAE (nervous system disorders) | 0 | 0 | 0 | 0 | 0 | 0 | 1 | 0 | 0 |
|  | SAE (respiratory, thoracic and mediastinal disorders) | 0 | 0 | 0 | 0 | 0 | 0 | 0 | 0 | 0 |
|  | SAE total | 0 | 0 | 9 | 5 | 6 | 0 | 14 | 0 | 3 |
|  | Total | 20 | 19 | 92 | 33 | 54 | 31 | 55 | 36 | 13 |
| Control group (n) | SAE (neoplasms) | 0 | 0 | 0 | 0 | 0 | 0 | 0 | 0 | 0 |
|  | SAE (immune system disorders) | 0 | 0 | 0 | 0 | 0 | 0 | 1 | 0 | 0 |
|  | SAE (infections and infestations) | 0 | 0 | 1 | 0 | 0 | 0 | 1 | 0 | 0 |
|  | SAE (gastrointestinal disorders) | 0 | 0 | 1 | 0 | 0 | 0 | 0 | 0 | 0 |
|  | SAE (general disorders) | 0 | 0 | 0 | 0 | 0 | 0 | 1 | 0 | 0 |
|  | SAE (hepatobiliary disorders) | 0 | 0 | 0 | 0 | 0 | 0 | 0 | 0 | 0 |
|  | SAE (reproductive system issues) | 0 | 0 | 0 | 0 | 0 | 0 | 0 | 0 | 0 |
|  | SAE (musculoskeletal and connective tissue disorders) | 0 | 0 | 0 | 0 | 0 | 0 | 1 | 0 | 1 |
|  | SAE (cardiac disorders) | 0 | 0 | 1 | 0 | 0 | 0 | 0 | 0 | 0 |
|  | SAE (blood and lymphatic system disorders) | 0 | 0 | 0 | 0 | 1 | 0 | 0 | 0 | 0 |
|  | SAE (nervous system disorders) | 0 | 0 | 0 | 0 | 0 | 0 | 2 | 0 | 0 |
|  | SAE (respiratory, thoracic and mediastinal disorders) | 0 | 0 | 0 | 1 | 0 | 0 | 0 | 0 | 0 |
|  | SAE total | 0 | 0 | 3 | 1 | 1 | 0 | 6 | 0 | 1 |
|  | Total (n) | 10 | 22 | 95 | 19 | 49 | 28 | 55 | 19 | 14 |

**
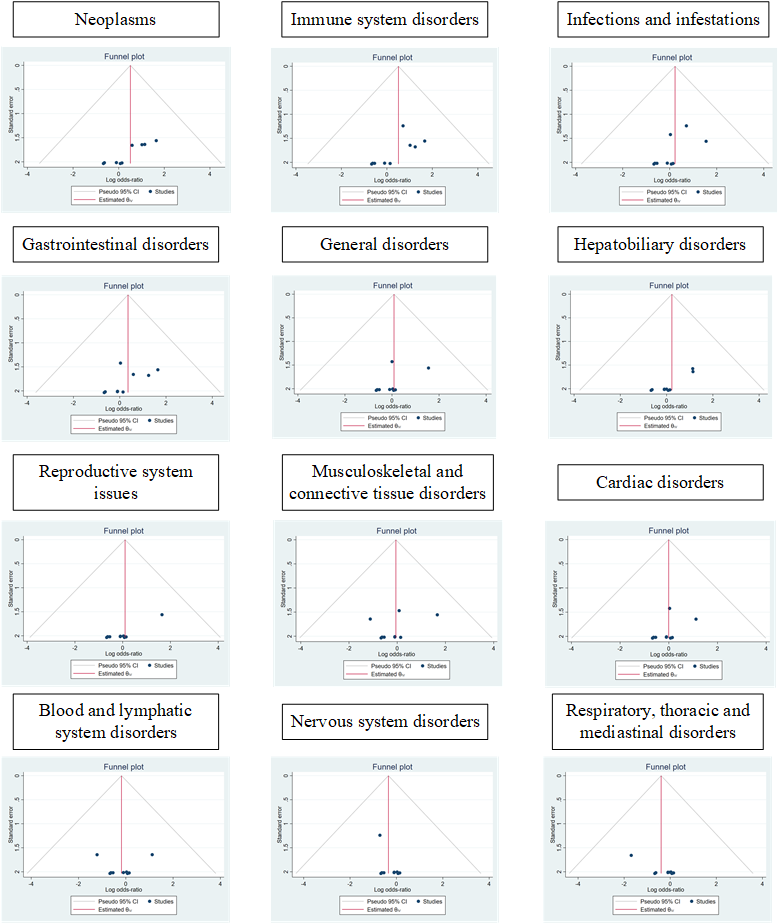
**

**Supplementary figure S1. Funnel plots of the publication bias of SAEs in different system disorders.**


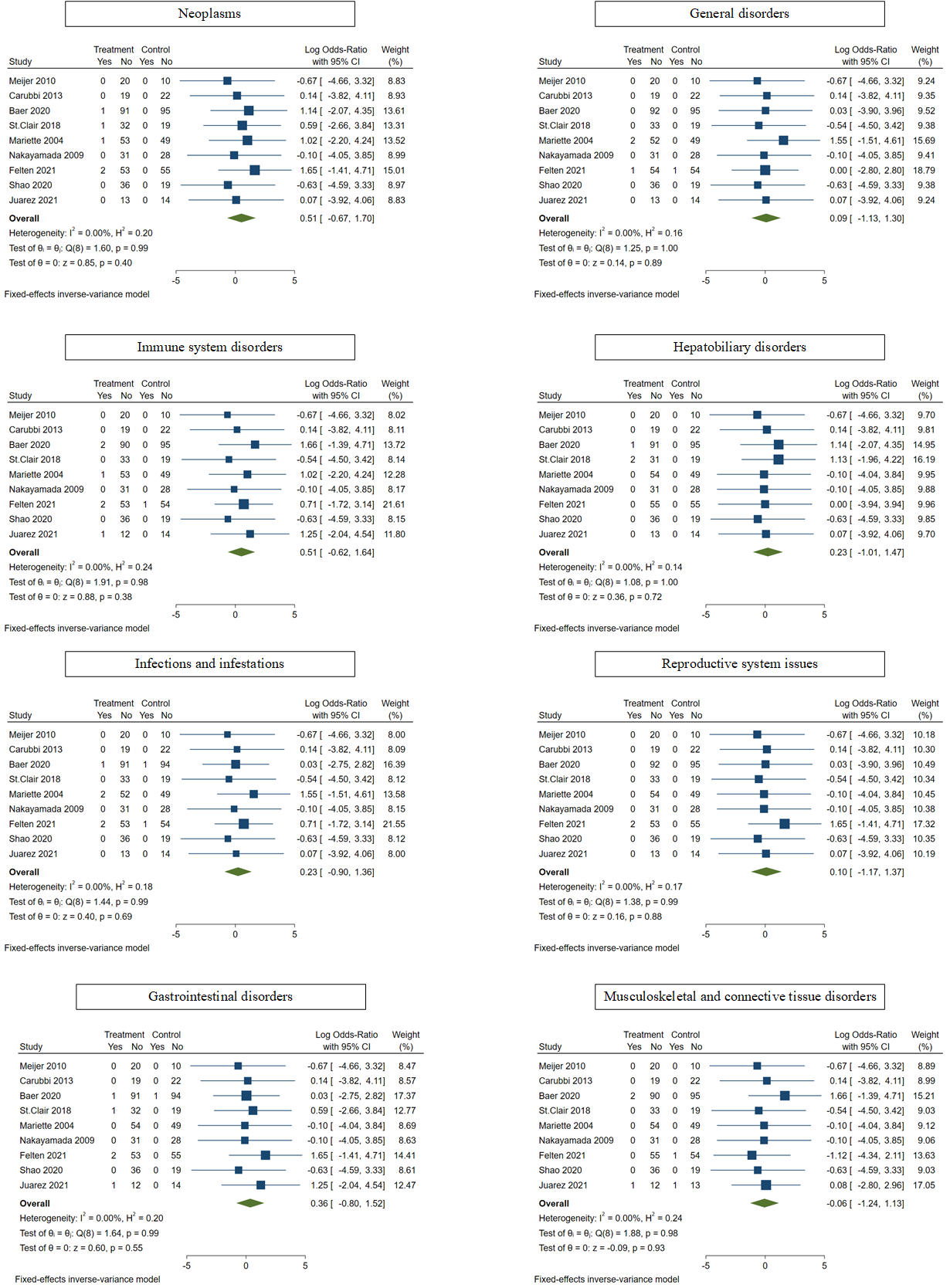


(continued)


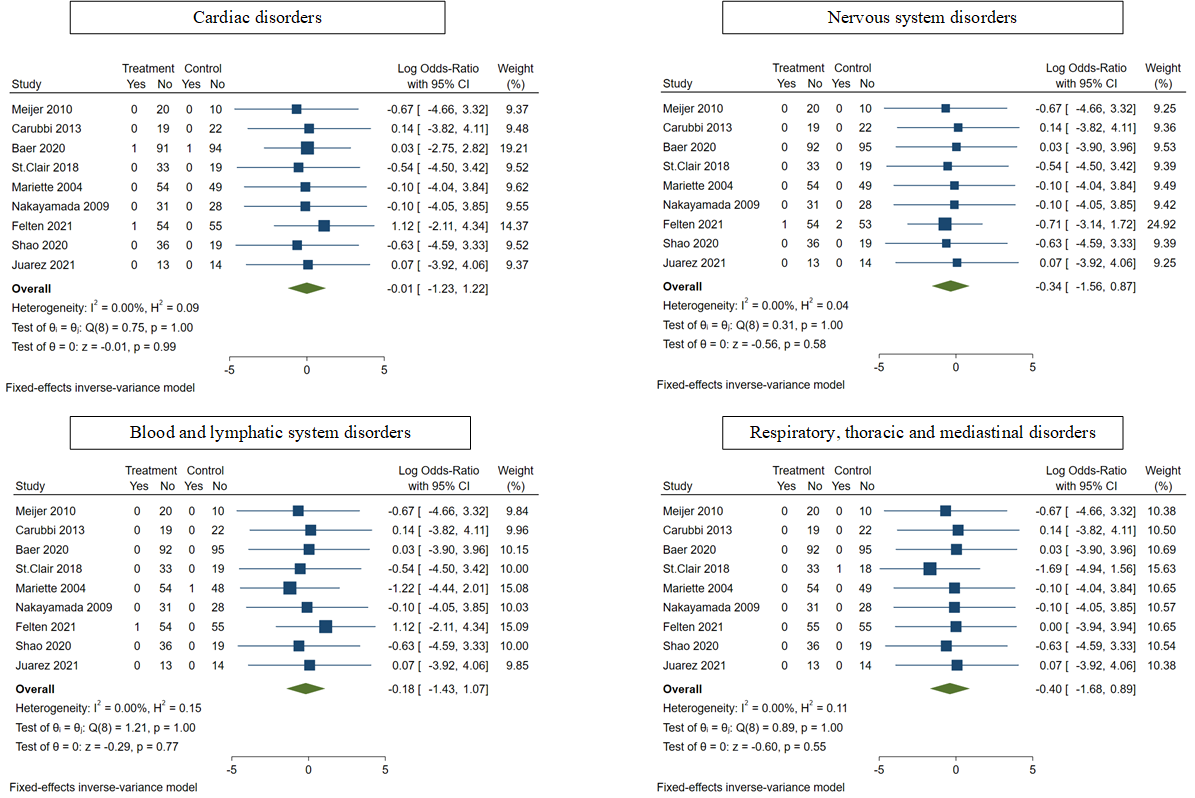


**Supplementary figure S2. Forest plot of studies comparing the intervention of biologics and control groups on pSS patients of SAEs in different system disorders.**
